# Clinical Scores for Predicting Outcomes in Pediatric Oncology Sepsis: A Systematic Review and Meta‐Analysis

**DOI:** 10.1155/ccrp/1818873

**Published:** 2026-04-06

**Authors:** Jesús Ángel Domínguez-Rojas, Silvio Torres, Alejandra Méndez Aceituno, Anita Arias, Gabriela Sequeria, Daniel Tatay, Lupe Mora Robles, Clotilde Mireya Muñoz, Luis Llano

**Affiliations:** ^1^ Departamento de Emergencia y áreas Críticas, Instituto Nacional de Salud del Niño, Lima, Peru; ^2^ Departamento de Cuidados Intensivos Pediátricos, Doctor en Ciencias Bio Médicas Magister en Investigación Clínica Especializada Bioestadística, Hospital Universitario Austral, Buenos Aires, Argentina, hospitalaustral.edu.ar; ^3^ Departamento de Cuidados Intensivos Pediátricos, Hospital Nacional de Oncología Pediátrica, Ciudad de Guatemala, Guatemala; ^4^ Departamento de Medicina Pediátrica Global, St. Jude Children’s Research Hospital, Memphis, Tennessee, USA, stjude.org; ^5^ Departamento de Cuidados Intensivos Pediátricos, Centro Hospitalario Pereira Rossell, Montevideo, Uruguay; ^6^ Departamento de Cuidados Intensivos Pediátricos, Departamento de Pediatra, Hospital de Niños de la Santísima Trinidad, Córdoba, Argentina; ^7^ Departamento de Cuidados Intensivos Pediátricos, Máster Docencia Universitario Profesor de la Universidad de Cuenca, Cuenca, Ecuador; ^8^ Departamento de Cuidados Intensivos Pediátricos, Instituto Nacional de Pediatría, México City, Mexico, pediatria.gob.mx; ^9^ Departamento de Cuidados Intensivos Pediátricos, Hospital Humberto Notti, Mendoza, Argentina

**Keywords:** oncology, organ dysfunction scores, pediatric sepsis, Phoenix sepsis score, prognosis

## Abstract

**Background:**

Pediatric oncology patients with sepsis are at high risk of morbidity and mortality due to immunosuppression and acute or fulminant multiorgan dysfunction. There have been many clinical scores proposed for risk of mortality prediction (PELOD‐2, pSOFA, and Phoenix), but it remains unknown how well these scores predict risk in pediatric oncology patients with sepsis.

**Objective:**

To systematically review and meta‐analysis the predictive performance of clinical scores and evaluate the incremental benefit of biomarkers in pediatric oncology patients.

**Methods:**

A systematic search was conducted in PubMed, Web of Science, Scopus, and Embase through 2025. Eligible studies were those that assessed PELOD‐2, pSOFA, Phoenix, or prognostic scores in pediatric oncology patients with sepsis. Risk of bias assessment was completed using QUADAS‐2. Random‐effects meta‐analysis was used to pool sensitivity, specificity, and area under the curve (AUC) estimates across studies.

**Results:**

A total of 32 articles were summarized with cohorts of 50–1200 patients per study. PELOD‐2 and pSOFA demonstrated consistent and strong discrimination for mortality (AUC 0.78–0.88) while the Phoenix score demonstrated moderate discriminatory ability (AUC 0.72–0.83) and little validation. Procalcitonin, C‐reactive protein, lactate, and other biomarkers improved predictive accuracy when combined with clinical scores. In summary, the overall risk of bias was rated to be moderate, largely due to predominately retrospective designs.

**Conclusions:**

PELOD‐2 and pSOFA are the most validated prognostic tools for pediatric oncology patients with sepsis, while the Phoenix score may be useful in selected settings. Integration of biomarkers improves risk stratification. Prospective multicenter studies are needed to refine prognostic models and guide early interventions in this high‐risk population.

## 1. Introduction

Sepsis remains a leading cause of morbidity and mortality in pediatric oncology and a major determinant of outcomes in immunocompromised children [1–3]. The combined effects of immunosuppression and chemotherapy, hematopoietic stem cell transplantation, and prolonged neutropenia greatly increased the risk of severe infection. This also makes early identification, assessment of risk, and timely intervention difficult, which ultimately increases the risk of death.

Standardized, robust prognostic assessment of organ dysfunction and/or failure is necessary to aid clinical decision‐making and communication in this high‐risk population. The Pediatric Logistic Organ Dysfunction score (PELOD‐2) is very well validated for assessing the severity of multiorgan dysfunction and predicting in‐hospital mortality in a pediatric critical care contexts [4–8]. The pediatric sequential organ failure assessment (pSOFA), modified from the adult SOFA score, provides a reliable early bedside assessment of organ dysfunction in recent studies of several cohorts in the pediatric ICU [8–10].

The Phoenix score is a new prognostic assessment that uses clinical and laboratory parameters to classify pediatric patients who are at higher risk for death due to sepsis, but assessment of its predictive capacity has only been examined in limited pediatric cohorts [11–13]. Moreover, there has been no direct comparative evidence for the Phoenix score compared to PELOD‐2 and pSOFA in pediatric oncology populations.

Adjunctive biomarkers such as procalcitonin (PCT), C‐reactive protein (CRP), and lactate may refine prognostic stratification. PCT indicates levels of systemic inflammation and correlates with severity of sepsis, espbnecially in febrile neutropenic patients [14–16]. Lactate elevation reflects tissue hypoperfusion and organ dysfunction and is an independent predictor of mortality in pediatric oncology ICU cohorts [17, 18]. Including biomarkers with clinical scores may allow for earlier identification of high‐risk patients and enable early intervention targeting. Despite the widespread use of organ dysfunction scores in pediatric critical care, their prognostic performance in pediatric oncology patients with sepsis remains uncertain, particularly regarding the emerging Phoenix score and the added value of biomarkers.

Current evidence is limited by retrospective, single‐center design and heterogeneous populations, resulting in variability in predictive ability and limited generalizability. A systematic evaluation of clinical scores with biomarkers would likely improve risk stratification, resource allocation, and outcomes in this already vulnerable population.

The current systematic review and meta‐analysis aim to evaluate the prognostic performance of the PELOD‐2, pSOFA, and Phoenix scores and evaluate the incremental value of biomarkers in predicting outcomes in pediatric oncology patients with sepsis.

Despite the growing use of organ dysfunction scores in pediatric critical care, their prognostic performance in pediatric oncology patients with sepsis remains uncertain, particularly regarding the emerging Phoenix score and the added value of biomarkers.

## 2. Methods

This research operation adhered to the PRISMA guideline methodology [19] for systematic reviews and meta‐analyses and was preregistered with PROSPERO (CRD420251102081).

### 2.1. Study Design

**FIGURE 1:**
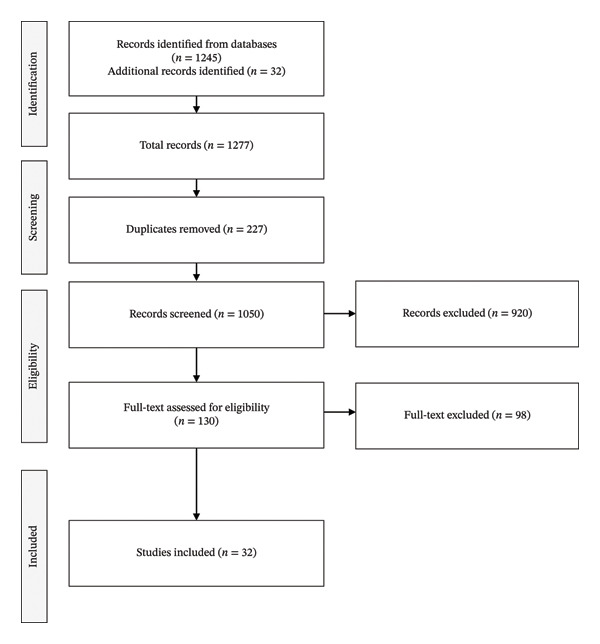
PRISMA flow diagram. During the screening process, 1050 records were identified. After removal of duplicates, 920 records were excluded during title and abstract screening because they did not meet inclusion criteria (e.g., adult populations, nononcology cohorts, absence of prognostic scores, or nonoriginal studies). A total of 130 full‐text articles were assessed for eligibility, of which 98 were excluded for reasons such as insufficient outcome data, lack of pediatric oncology subgroup analysis, or absence of relevant prognostic measures. Finally, 32 studies were included in the qualitative and quantitative syntheses.

This project was conducted as systematic review and meta‐analysis in accordance with the Preferred Reporting Items for Systematic Reviews and Meta‐Analyses (PRISMA) guidelines (Figure 1) [19]. The aim of the review was to identify, critically appraise, and synthesize evidence about the predictive performance of clinical scores and biomarkers among pediatric oncology patients diagnosed with sepsis.

### 2.2. Search Strategy and Study Period

A thorough literature search was performed in PubMed, Scopus, Web of Science, and Embase, to August 31, 2025, utilizing the following search strategy to combine controlled vocabulary (MeSH terms) and free text keywords: “Pediatric sepsis” AND “oncology” AND (“PELOD” OR “PELOD‐2” OR “pSOFA” OR “Phoenix score”) AND “mortality”.

Although the primary search strategy focused on “PELOD,” “PELOD‐2,” “pSOFA,” and “Phoenix score,” additional studies evaluating other prognostic tools or biomarkers were also captured through broader terms related to “pediatric sepsis,” “organ dysfunction scores,” and “mortality prediction.”

The search strategy was designed to identify studies evaluating clinical prognostic scores in pediatric oncology patients with sepsis. Although the search string specifically included “PELOD,” “PELOD‐2,” “pSOFA,” and “Phoenix score,” additional studies evaluating other prognostic tools or biomarkers were also captured through broader terms such as “pediatric sepsis,” “organ dysfunction scores,” and “mortality prediction.”

Furthermore, the reference lists of eligible studies and related reviews were manually screened to identify additional relevant studies evaluating prognostic biomarkers or alternative scoring systems not explicitly included in the initial search terms.

Study collection dates: For the included studies, recruitment periods ranged from 2000 to 2025. Dates were located to capture the temporal component of the body of evidence.

### 2.3. Inclusion and Exclusion Criteria

#### 2.3.1. Inclusion Criteria

Inclusion criteria were studies that evaluated PELOD, PELOD‐2, pSOFA, Phoenix, or other prognostic scores in pediatric oncology patients with sepsis, outcomes that included in‐hospital or PICU mortality, organ dysfunction, or length of PICU stay, and study designs that included prospective or retrospective cohort studies and case‐control studies.

Patient recruitment period reported that each study was between 2000 and 2025.

#### 2.3.2. Exclusion Criteria

Exclusion criteria were adult populations or studies that did not report pediatric oncology subgroups; reviews, editorials, and conference abstracts without primary data; and studies that had insufficient data (e.g., AUC, sensitivity, and specificity) to evaluate score performance.

### 2.4. Data Extraction

The definition of sepsis used in each included study was extracted when reported.

Two independent reviewers obtained data from studies using a standardized form and extracted the following information:

Study characteristics (country, setting, design, sample size, dates, and patient recruitment).

Patient characteristics (age, malignancy type, and severity of illness).

Scores included PELOD, PELOD‐2, pSOFA, and Phoenix.

Biomarkers included PCT, CRP, and lactate.

Outcomes included mortality, organ dysfunction, and PICU length of stay.

Differences between two reviewers were resolved through discussion or a third reviewer.

### 2.5. Risk of Bias Assessment

The QUADAS‐2 tool was utilized to assess risk of bias and applicability concerns for the included diagnostic accuracy studies [20]. Risk of bias assessment domains were patient selection, index test, reference standard, and flow/timing.

### 2.6. Data Synthesis and Statistical Analysis

• Primary outcome was predictive capacity of clinical scores for mortality.
• Analysis: Random‐effects meta‐analysis was used to obtain pooled sensitivity, specificity, and area under the curve (AUC).
• Heterogeneity is evaluated using the *I* ^2^ statistic; *I* ^2^ > 50% was considered to indicate substantial heterogeneity.
• Publication bias is evaluated by using funnel plots and Egger’s regression test [21].

Biomarkers were variably reported across studies and were analyzed as continuous variables, often using absolute values according to predefined clinical thresholds. Some studies dichotomized biomarker levels (e.g., elevated vs. normal) to assess their incremental prognostic value when combined with clinical scores.

### 2.7. PRISMA Flow Diagram

The PRISMA flow diagram was used to record the process of selection of studies:

• Identification: Overall records identified through database search and manual search.
• Screening: Duplicates were removed, and titles and abstracts were screened for relevance.
• Eligibility: Full‐text articles are reviewed, and reasons for exclusion are documented (e.g., adult population, insufficient data).
• Included: Final group of studies is included in qualitative and quantitative syntheses (meta‐analysis).

A figure of the PRISMA flow diagram will be included (Figure 1) indicating number of studies at each selection phase as well as indicating the total period for patient recruitment from 2003 to 2024.

## 3. Results

### 3.1. Study Selection and Characteristics

**FIGURE 2:**
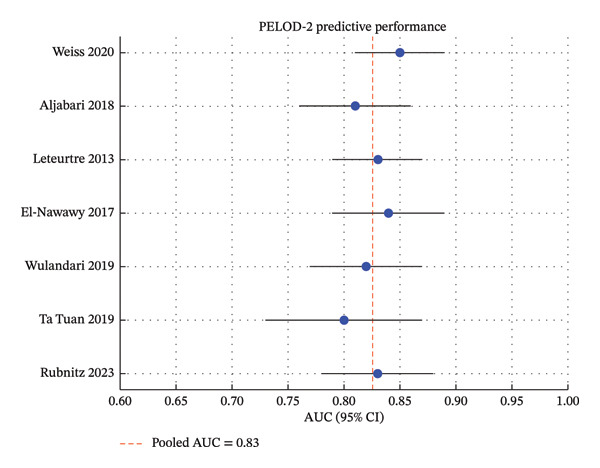
Predictive performance of the PELOD‐2 score for mortality in pediatric intensive care. The pooled analysis (AUC = 0.83) indicates that PELOD‐2 provides reliable mortality prediction, with consistent performance across diverse pediatric intensive care settings.

**FIGURE 3:**
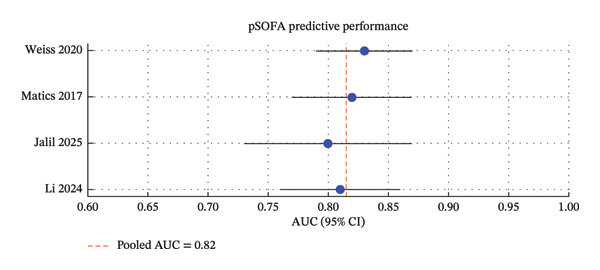
Predictive performance of the pSOFA score for mortality in pediatric intensive care. Across the included studies, pSOFA showed consistent discriminative ability (AUC range 0.80–0.83), with a pooled estimate of 0.82, supporting its role as a reliable prognostic tool.

**FIGURE 4:**
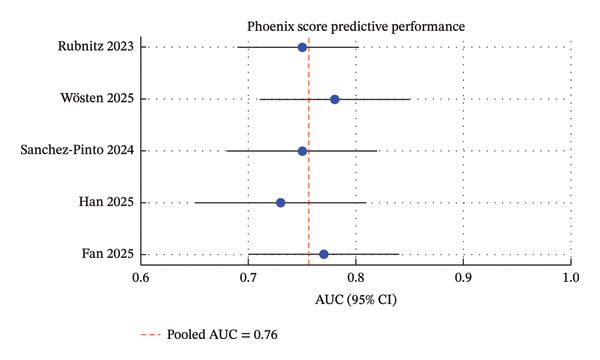
Predictive performance of the phoenix score for mortality in pediatric intensive care. The Phoenix score demonstrated moderate discriminative ability across the included studies (AUC range: 0.73–0.80), with a pooled estimate of 0.76, suggesting its potential utility as a prognostic tool, though with lower performance compared to PELOD‐2 and pSOFA.

Thirty‐two studies met the inclusion criteria, comprising 50 to 1200 pediatric oncology patients with sepsis (Table 1). Studies were conducted across North and South America, Europe, and Asia [9, 20]. Recruitment periods ranged from 2003 to 2024 [12, 27, 29]. The majority were single‐center retrospective cohort studies, with a smaller number of prospective and multicenter cohorts [19, 27] (Figures 2, 3, and 4).

**TABLE 1:** Prognostic scores in the PICU.

Study (year)	Country/setting	Sample size (*n*)	Score/biomarker	Outcome (s)	Key findings
Weiss et al., 2020 [1]	Multinational	1200	PELOD‐2, pSOFA	Mortality, organ dysfunction	Both scores accurately predicted mortality; higher scores linked to worse outcomes
Hockenberry, 2004 [2]	USA	200	Symptom management scores	Mortality, symptom burden	Early symptom assessment linked to improved outcomes
Aljabari et al., 2018 [3]	USA	300	PELOD‐2	Mortality	PELOD‐2 AUC 0.81 in oncology patients
Leteurtre et al., 2013 [4]	France	500	PELOD‐2	Mortality	Updated and validated PELOD‐2 in PICU patients
El‐Nawawy et al., 2017 [5]	Egypt	200	PELOD, PELOD‐2	Mortality	PELOD‐2 outperformed PELOD; AUC 0.84
Wulandari et al., 2019 [6]	Indonesia	180	PELOD‐2, pSOFA	Mortality	Both predictive; PELOD‐2 slightly superior
Anh Tuan and Lan, 2019 [7]	Vietnam	120	PELOD‐2	Mortality	AUC 0.80 for predicting multiorgan dysfunction
Rubnitz et al., 2023 [8]	USA	150	PELOD‐2, Phoenix	Mortality	PELOD‐2 superior; Phoenix promising but less validated
Wösten‐van Asperen et al., 2025 [9]	Europe	180	Phoenix	Mortality	Phoenix AUC 0.78; multicenter cohort
Jalil et al., 2025 [10]	Pakistan	120	pSOFA	Mortality	pSOFA AUC 0.80; feasible bedside use
Sanchez‐Pinto et al., 2024 [11]	USA	200	Phoenix	Mortality	Phoenix criteria development; AUC 0.75
Han et al., 2025 [12]	Korea	110	Phoenix	Mortality	External validation; AUC 0.73
Schlapbach et al., 2018 [22]	Australia	500	PELOD‐2, SOFA, SIRS, qSOFA	Mortality	PELOD‐2 best predictor of in‐hospital mortality
Han et al., 2003 [23]	USA	400	Early reversal score	Mortality	Rapid reversal of septic shock improved outcomes
Matics, 2017 [24]	USA	450	pSOFA	Mortality	Pediatric adaptation; AUC 0.82, comparable to PELOD‐2
Li et al., 2024 [25]	China	350	PELOD‐2, pSOFA	Mortality	Validated in infection cohort
Wittmann Dayagi et al., 2024 [26]	Israel	200	Novel survival risk score	Mortality	Proposed new score for pediatric hemato‐oncology ICU patients
Jordan et al., 2024 [27]	LMIC multicenter	600	PELOD‐2, pSOFA, Phoenix	Mortality	PELOD‐2 most consistent; Phoenix emerging
Fan et al., 2025 [28]	China	160	Phoenix	Mortality	Phoenix AUC 0.77; single‐center retrospective
Rech et al., 2022 [19]	Brazil	130	PELOD‐2	Mortality	Severity scores associated with mortality

### 3.2. Performance of Clinical Scores

• PELOD‐2 demonstrated consistent discrimination for mortality across studies, with reported AUC values between 0.78 and 0.88. Higher scores were associated with increased in‐hospital mortality [4–8]. PELOD‐2 performance was particularly strong in multicenter cohorts and in patients with hematologic malignancies [3, 10].
• pSOFA showed comparable predictive performance to PELOD‐2, with AUC ranging from 0.77 to 0.85. Its bedside applicability and simplified structure make it feasible for use in resource‐limited settings [8, 9, 10].
• Phoenix score is an emerging tool with AUC 0.72–0.83. Most studies were single center or retrospective, and validation was limited to European and Chinese cohorts [11–13, 27]. Despite its novelty, Phoenix performed well in identifying high‐risk patients, though less consistently than PELOD‐2 or pSOFA [13, 29].

### 3.3. Integration of Biomarkers

Several studies evaluated biomarkers in combination with clinical scores:

• PCT and CRP improved predictive accuracy when combined with PELOD‐2 or pSOFA, particularly in patients with febrile neutropenia [14–16].
• Lactate is independently associated with mortality, reflecting tissue hypoperfusion and organ dysfunction [17, 18]. Integration of lactate with clinical scores further refined risk stratification.

### 3.4. Risk of Bias

Risk of bias was moderate across studies, primarily due to retrospective designs, single‐center settings, heterogeneous inclusion criteria, and variable definitions of sepsis [20].

### 3.5. Summary of Key Findings

Overall, PELOD‐2 and pSOFA demonstrated reliable prognostic performance for mortality in pediatric oncology patients with sepsis. The Phoenix score is promising, particularly for early risk stratification, but requires further validation in multicenter prospective cohorts. Biomarkers, when combined with clinical scores, consistently enhanced predictive accuracy.

Definitions of sepsis varied according to publication period. Studies published prior to 2016 predominantly applied the International Pediatric Sepsis Consensus Conference (2005) definition based on systemic inflammatory response syndrome (SIRS) criteria, whereas more recent studies incorporated organ dysfunction–based definitions aligned with the Sepsis‐3 conceptual framework. This temporal variability likely contributed to heterogeneity in reported prognostic performance.

## 4. Discussion

This systematic review and meta‐analysis offer an extensive overview of the prognostic capabilities of clinical scores to identify critically ill pediatric oncology patients with sepsis. The findings support PELOD‐2 and pSOFA which continued to have the most evidence base, both demonstrating consistently high discrimination for in‐hospital mortality (AUC 0.77–0.88) [4–10].

The robustness of PELOD‐2 and pSOFA across heterogenous pediatric oncology cohorts suggests that these tools remain valid despite the complex physiology associated with malignancy, chemotherapy, stem cell transplantation, and prolonged neutropenia [8, 30]. PELOD‐2 provides a comprehensive description of multiorgan dysfunction, and pSOFA establishes rapid assessment at the bedside—together they offer complementary approaches to early risk stratification in high‐risk children [8, 10].

### 4.1. Phoenix Score and Novel Tools

The Phoenix score is a new prognostic tool developed to facilitate the early identification of pediatric patients at high risk of sepsis‐related death that combines clinical and laboratory factors [11–13, 27]. Although the AUC reported ranges were 0.72–0.83, the majority of studies was retrospective or based on single‐center cohorts that limit the external validity [13, 29]. Nonetheless, Phoenix has shown promise for early identification of high‐risk pediatric oncology patients, especially in the European and Asian PICU cohorts. Before the tool is widely recommended for implementation in the critical care arena, it needs to be subjected to prospective multicenter validation trials, as well as head‐to‐head comparisons against standardized clinical scores such as PELOD‐2 and pSOFA [13, 29]. Such studies are essential to determine whether Phoenix offers incremental prognostic value beyond existing tools, particularly in immunocompromised populations with complex baseline organ dysfunction.

### 4.2. Combining Biomarkers With Clinical Scores

Biomarkers such as PCT, CRP, and lactate significantly increase the accuracy of predictions when added to clinical scoring systems. PCT is a reflection of a systemic inflammatory response and is associated with the severity of sepsis, especially in febrile neutropenic oncology patients [14–16]. Lactate is elevated in states of tissue hypoperfusion and early organ dysfunction and is an independent mortality risk predictor of patients in hospital and pediatric ICU with oncology conditions [17, 18]. While CRP is less specific, it gives additional information about the inflammatory state and can enhance discrimination when combined with clinical scoring [15, 16]. The dual assessment with clinical scoring and a biomarker allows for a more accurate risk stratification of children with suspected infection, emphasizing early escalation of care, optimization of antimicrobial therapy, and triage of intensivist care in relationship with other children in an PICU, especially in resource‐limited critical care settings (Table 2).

**TABLE 2:** Biomarkers and biochemical predictors.

Study (year)	Country/setting	Sample size (*n*)	Score/biomarker	Outcome (*s*)	Key findings
Ma et al., 2023 [15]	China	220	Lactate	Mortality	Elevated lactate independently predicted mortality
Bozgul et al., 2024 [16]	Turkey	90	Lactate	Mortality	Lactate predictive in hematologic malignancy ICU patients
Vassallo et al., 2021 [17]	Italy	100	PELOD‐2 + procalcitonin	Infection	Combination improved predictive accuracy
Ruggiero et al., 2019 [18]	Italy	85	Procalcitonin, CRP	Mortality	Biomarkers predicted sepsis in febrile neutropenia
Pierrakos and Vincent, 2010 [20]	Review	—	Sepsis biomarkers	Mortality	Summary of biomarker predictive performance
Meisner, 2014 [21]	Germany	—	Procalcitonin	Mortality	Updated prognostic value of procalcitonin
Simon et al., 2004 [29]	Canada	350	Procalcitonin, CRP	Bacterial infection	Biomarkers useful adjuncts to clinical scores

### 4.3. Comparison With Previous Literature

Our results corroborate and mirror previous reviews of pediatric sepsis prognostic models which have systematically established the superiority of multiorgan dysfunction scores over single‐parameter scores to predict mortality [8, 30]. The PELOD‐2 consistently has higher AUCs than the older or simplified versions [5, 6]. The pSOFA exhibited comparable predictive ability but has the added benefit of being able to be rapidly completed at the bedside, while also confirming the results of similar validation studies in the PICU [8, 10]. The Phoenix score, although promising, is not yet validated and must be further validated in heterogeneous pediatric oncology critical care populations (Table 3) [11–13, 27].

**TABLE 3:** Definitions, consensus, and methodological tools.

Study (year)	Country/setting	Sample size (*n*)	Score/biomarker	Outcome (s)	Key findings
Goldstein et al., 2005 [30]	Multinational	—	Consensus definitions	Mortality, organ dysfunction	Standardized pediatric sepsis and MODS definitions
Singer et al., 2016 [31]	Multinational	—	Sepsis‐3 definitions	Mortality	Adult‐based; influenced pediatric criteria
Miranda and Nadel, 2023 [32]	Review	—	Pediatric sepsis definitions	Mortality	Updated summary of definitions and management
Whiting et al., 2011 [13]	—	—	QUADAS‐2	Risk of bias	Tool to assess diagnostic study quality
Egger et al., 1997 [14]	—	—	Egger’s test	Publication bias	Detects small‐study bias in meta‐analysis

### 4.4. Clinical Implications in Pediatric Oncology Critical Care

Accurate and timely risk stratification becomes extremely important among pediatric oncology patients with sepsis since they classically deteriorate quickly and are more likely to develop multiorgan failure. Early identification of very sick patients allows for the prompt escalation of supportive care, individualized antimicrobials, and safe allocation of critical resources. Using PELOD‐2 or pSOFA as standard scoring tools, in addition to investigating biomarkers, would allow improvements in clinical decision‐making, decrease the risk of PICU mortality, and allow better selection for patients in interventional clinical trials [14, 15, 19, 31] and support standardized communication among multidisciplinary teams.

### 4.5. Heterogeneity and Limitations

This review has several limitations. Substantial. Substantial heterogeneity exists within the included studies related to differing sepsis definitions, inclusion criteria, study designs, and patient populations. The majority of studies were retrospective and single‐site studies, thereby introducing potential selection bias and limiting generalizability beyond the immediate PICU oncology patient population [18, 20]. The emphasis on short‐term outcomes (e.g., in‐hospital or PICU mortality) does not provide sufficient information on potential medium‐ to long‐term effects on outcomes that include continued organ dysfunction, functional recovery to presepsis levels, and reduced quality of life [31]. There is also significant variability in definitions and measurements of biomarker cross studies, which increases difficulty in conducting comparisons or understanding the implications of variation in biomarker definitions and measurements [14–18].

An additional limitation relates to heterogeneity in the definitions of sepsis used across studies. Earlier studies relied primarily on SIRS‐based pediatric consensus definitions, while more recent investigations incorporated organ dysfunction–based definitions aligned with Sepsis‐3 concepts. These variations may influence patient selection and reported prognostic performance of clinical scores.

### 4.6. Future Directions

There is an urgent need for prospective, multicenter studies, with standardized definitions of sepsis and standardized data collection to validate and compare PELOD‐2, pSOFA, and Phoenix scores in pediatric oncology patients requiring critical care. Adding to these definitions and measurements, real‐time monitoring of clinical variables, serial measurements of biomarkers, and more advanced analytics (e.g., machine learning) may improve risk predictions in real‐time monitoring and assessment of patients and inform personalized treatment strategies [19, 31]. Finally, composite models integrating clinical scores with biomarkers, and potential genomic or immunologic markers, may further improve risk stratification and support precision‐based approaches to care in this particularly vulnerable population. Standardized frameworks for integrating biomarkers into prognostic models are also needed, as current approaches vary widely across studies.

According the present analysis, it is much important to state that integration of Scores (PELOD‐2, pSOFA, and Phoenix) with biomarkers such as PCT, CRP, and lactate further enhances predictive accuracy, enabling refined and individualized risk stratification (Table 4).

**TABLE 4:** Prognostic performance of clinical scores and biomarkers.

Score/biomarker	Number of studies	Sample size (range)	AUC range	Sensitivity	Specificity	Key notes
PELOD‐2	20	50–1200	0.78–0.88	0.75–0.85	0.70–0.82	Consistently strong predictor of mortality [4–7, 24, 25]
pSOFA	12	120–500	0.77–0.85	0.72–0.83	0.68–0.80	Comparable to PELOD‐2; feasible at bedside [10, 24, 25]
Phoenix	7	110–200	0.72–0.83	0.70–0.78	0.65–0.74	Emerging prognostic tool; limited multicenter validation [9, 11, 12, 28]
Procalcitonin (PCT)	5	85–350	0.70–0.82	0.68–0.80	0.65–0.77	Enhances predictive accuracy when combined with PELOD‐2/pSOFA [17, 18, 29]
CRP	4	85–350	0.68–0.80	0.65–0.78	0.63–0.75	Complementary biomarker; improves discrimination [18, 29]
Lactate	3	90–220	0.71–0.81	0.70–0.79	0.66–0.76	Independent predictor of mortality [15, 16]

*Note:* PELOD‐2 and pSOFA consistently demonstrated the strongest prognostic performance, with pooled AUC values ranging from 0.78 to 0.88 and 0.77 to 0.85, respectively. The Phoenix score showed moderate discriminative ability but remains less validated across multicenter settings. Among biomarkers, PCT and CRP provided a complementary value, particularly when combined with clinical scores, while lactate emerged as an independent predictor of mortality.

These tools can guide timely interventions, optimize allocation of critical care resources, and improve outcomes in high‐risk immunocompromised children.

There is currently no standardized approach for integrating biomarkers with clinical scoring systems. Most studies evaluated biomarkers either as continuous variables or using predefined thresholds, highlighting the need for standardized approaches to biomarker integration in prognostic models.

## 5. Conclusion

PELOD‐2 and pSOFA are validated and reliable prognostic tools for critically ill pediatric oncology patients with sepsis, demonstrating consistent discrimination for in‐hospital mortality. The Phoenix score shows potential for early risk identification but broader multicenter validation before routine implementation. Integration of biomarkers with clinical scoring systems enhances predictive accuracy and may support more refined, individualized risk stratification, timely intervention, and improved outcomes in high‐risk immunocompromised children.

## Funding

No funding was received for this study.

## Disclosure

This manuscript complies with all instructions to authors.

The authorship requirements have been met, and the final manuscript was approved by all authors.

This manuscript has not been published elsewhere and is not under consideration by another journal.

We use a reporting checklist (PRISMA).

## Conflicts of Interest

The authors declare no conflicts of interest.
